# Continuous non-invasive vs. invasive arterial blood pressure monitoring during neuroradiological procedure: a comparative, prospective, monocentric, observational study

**DOI:** 10.1186/s13741-024-00442-3

**Published:** 2024-07-22

**Authors:** Xavier Chapalain, Thomas Morvan, Jean-Christophe Gentric, Aurélie Subileau, Christophe Jacob, Anna Cadic, Anaïs Caillard, Olivier Huet

**Affiliations:** 1 Department of Anesthesiology and Surgical Intensive Care, University and Regional Hospital Centre Brest, Boulevard Tanguy Prigent, Brest, Cedex 29609 France; 2 Laboratoire ORPHY, Université de Bretagne Occidentale, Brest, France; 3 Department of Neuroradiology, University and Regional Hospital Centre Brest, Brest, France; 4 Laboratoire GETBO, UMR 1304, Université de Bretagne Occidentale, Brest, France

**Keywords:** Blood pressure, Hemodynamic monitoring, Intraoperative, Neuroradiology

## Abstract

**Background:**

In the perioperative setting, the most accurate way to continuously measure arterial blood pressure (ABP) is using an arterial catheter. Surrogate methods such as finger cuff have been developed to allow non-invasive measurements and are increasingly used, but need further evaluation. The aim of this study is to evaluate the accuracy and clinical concordance between two devices for the measurement of ABP during neuroradiological procedure.

**Methods:**

This is a prospective, monocentric, observational study. All consecutive patients undergoing a neuroradiological procedure were eligible. Patients who needed arterial catheter for blood pressure measurement were included. During neuroradiological procedure, ABP (systolic, mean and diatolic blood pressure) was measured with two different technologies: radial artery catheter and Nexfin. Bland-Altman and error grid analyses were performed to evaluate the accuracy and clinical concordance between devices.

**Results:**

From March 2022 to November 2022, we included 50 patients, mostly ASA 3 (60%) and required a cerebral embolization (94%) under general anaesthesia (96%). Error grid analysis showed that 99% of non-invasive ABP measures obtained with the Nexfin were located in the risk zone A or B. However, 65.7% of hypertension events and 41% of hypotensive events were respectively not detected by Nexfin. Compared to the artery catheter, a significant relationship was found for SAP (*r*^2^ = 0.78) and MAP (*r*^2^ = 0.80) with the Nexfin (*p* < 0.001). Bias and limits of agreement (LOA) were respectively 9.6 mmHg (− 15.6 to 34.8 mmHg) and − 0.8 mmHg (− 17.2 to 15.6 mmHg), for SAP and MAP.

**Conclusions:**

Nexfin is not strictly interchangeable with artery catheter for ABP measuring. Further studies are needed to define its clinical use during neuroradiological procedure.

**Trial registration:**

Clinicaltrials.gov, registration number: NCT05283824.

**Supplementary Information:**

The online version contains supplementary material available at 10.1186/s13741-024-00442-3.

## Introduction

There are growing evidences about the risk of hypertensive and hypotensive events during the perioperative period, making arterial blood pressure (ABP) probably one of the most important parameters to optimize (Abbott et al. 2018; Halvorsen et al. 2022; Salmasi et al. 2017). During major surgery, a low ABP and its variability have been associated with a higher risk of mortality, myocardial infarction, stroke and acute kidney injury (Gregory et al. 2021; Mascha et al. 2015; Salmasi et al. 2017). High blood pressure may also be harmful and may increase the risk of perioperative haemorrhage, cerebrovascular events and myocardial infarction (Abbott et al. 2018; Reich et al. 2002). Recently, two expert consensus statements emphasized the need of strict blood pressure control to improve perioperative care (McEvoy et al. 2019; Sessler et al. 2019).

Neuroradiological procedures can be considered a high-risk procedure, and it has been reported that hypotensive events might be associated with brain damage (Collette et al. 2021; Maïer et al. 2019; Valent et al. 2020). Therefore, a reliable and accurate ABP measurement to maintain cerebral perfusion is recommended (Lidington et al. 2021; Muldoon and Appleby 2020). In a recent French consensus statement, experts emphasized the need of a tight ABP control following thrombectomy (Quintard et al. 2023). Intermittent ABP measurement with an automated arm cuff remains the most used device in the operating room, and it has been recommended by the American Society of Anaesthesiologists as a standard of care during anaesthesia (Fellahi et al. 2021; Halvorsen et al. 2022; Vallet et al. 2013). However, the gold standard for continuous ABP measurement remains the placement of an arterial catheter in the radial or the femoral artery. This technic may expose the patient to local complications such as bleeding, arterial thrombosis, aneurysm or infection (Scheer et al. 2002). Even if invasive method had some advantages, the catheterization of radial or femoral artery may be difficult and delay the procedure (Saver 2006).

Recently, new continuous non-invasive ABP monitoring devices have been developed. Among them, the Nexfin technology is able to continuously measure ABP based on two principles: the volume-clamp method and the photoplethysmography technology.

In the operating room, some studies have shown reliable measures between the Nexfin and invasive measurement with acceptable agreement for ABP (Lu and Dalia 2021; Mukai et al. 2021; Schumann et al. 2021; Wang et al. 2022). To our best knowledge, there is only one observational monocentric study which investigated the accuracy of this new device during neuroradiological procedure (Bugarini et al. 2021). There are only a few studies that investigated the ‘clinical concordance’ of non-invasive ABP measurement (Bugarini et al. 2021; Kho et al. 2022; Yahagi et al. 2022).

In the current study, we evaluated the clinical relevance and accuracy of ABP provided by Nexfin, non-invasive continuous measures, compared to those provided by an arterial catheter during elective or emergency neuroradiological procedure.

## Materials and methods

### Study design and population

This is a prospective, observational study conducted in the Department of Anaesthesiology and Surgical Intensive Care Unit at Brest University Hospital Centre. All adult patients admitted in the operating room for an elective or emergent neuroradiological procedure and needed the placement of an arterial catheter to measure ABP were eligible. Exclusion criteria were as follows: contraindication of the placement of arterial catheter or digital cuff (Raynaud syndrome or Buerger syndrome), pregnant women and refusal to participate. The study protocol was registered on clinicaltrial.gov (registration number: NCT05283824, date of registration: 17th March 2022).

### Ethics

Ethical approval for this study was provided by the Ethic Committee of Est I (Ethical Committee N°2021-A02255-36) on 16th December 2021. This study was conducted in accordance with the principles of the Declaration of Helsinki. Written informed consent was provided to all eligible patients at inclusion.

### Perioperative management

Neuroradiological procedures done under general anaesthesia had the same anaesthetic protocol, consisting in a continuous propofol infusion, remifentanil target-controlled infusion and a neuromuscular blocker agent if needed. When performed under local anaesthesia, continuous remifentanil target-controlled infusion was used if deemed necessary. Haemodynamic management was performed using boluses of ephedrine and intraoperative continuous infusion of norepinephrine if deemed necessary by the physician in charge. The mean arterial pressure target was left at the discretion of the physician in charge. At the end of the procedure, all patients were transferred to the recovery room and then admitted in neuro-intensive care unit.

### ABP measurement

ABP was monitored as follows:

- First, a radial arterial catheter was inserted before the beginning of the procedure on the same arm (arterial catheter from Arrow®: length of 5 cm, diameter of 20 Ga). The invasive ABP signal was recorded at a frequency of 12 Hz using our standard equipment (Philips Intellivue®). All pressure signals were zeroed at the midaxillary line after the placement of the catheter. In routine, radial artery was considered by anaesthesiolgist as the reference measure for ABP in the operating room.
- Second, a continuous non-invasive ABP was measured on the same arm with the Nexfin device with an appropriate digital cuff (Clearsight® from the Edwards Lifesciences® Corporation, Irvine, California, USA). The Heart Reference System sensor was used in order to correct the hydrostatic pressure difference between the finger and the heart. Afterwards, the finger cuff and the heart reference system were connected to a wrist-processing unit that was in turn connected to the Hemosphere® clinical platform (Edwards Lifesciences®).

### Data collection

We collected demographic characteristics, comorbidities, ASA score, type of neuroradiological procedure and type of anaesthesia. We concomitantly recorded systolic, diastolic and mean arterial blood pressure (SAP, DAP and MAP) every 15 min from the beginning to the end of the procedure with both non-invasive (Nexfin) and invasive (radial artery catheter) devices. We also collected a set of measurements immediately before and after the use of norepinephrine.

### Objectives

The main objectives of our study were as follows: (i) to evaluate clinical relevance of ABP measures provided by non-invasive (Nexfin) and invasive device (radial artery catheter), (ii) to compare accuracy of ABP measures provided by non-invasive (Nexfin) in relation to gold standard (radial artery catheter).

### Statistical analysis

Patient characteristics were summarized using mean, standard deviation (SD) for continuous variables. Number and percentage were used for categorical variables.

Clinical relevance of the differences between each device was assessed using an error grid analysis (Saugel et al. 2018). Error grid analysis assigns a specific risk level value, ranking from A to E, for each pair of measured arterial pressures (Saugel et al. 2018). The risk levels were quantified for SAP and MAP by consensus among 25 international experts (Saugel et al. 2018). The clinical relevance of the difference between invasive and non-invasive monitoring is illustrated by the proportion of measurements in each risk level. We have also confronted ABP measures obtained with Nexfin in case of hypertension (defined by a SAP > 140 mmHg with radial artery) and hypotensive (defined by a MAP < 65 mmHg with radial artery) events. Hypertensive hypotensive events were defined according to recent international guidelines (Mancia et al. 2023; Sessler et al. 2019).

The values for MAP and SAP were compared for both devices. To visualize relationship between the ABP obtained with the reference method (radial artery catheter) and with non-invasive device (Nexfin), the arterial pressure values were plotted on a scatterplot, and the associated regression lines were displayed in scatter for MAP and SAP separately. The estimated correlation was calculated using Pearson correlation. Correlation values ≤ 0.20 are poor, while values ≥ 0.80 are excellent.

To assess the agreement between the two devices, we performed a Bland-Altman analysis and calculated the mean differences between the two non-invasive devices and the reference method (bias) and the 95% limits of agreement. We defined a priori the rules to evaluate the accuracy and precision of measures according to the Association for the Advancement of Medical Instrumentation (AAMI) 2019 guidelines (Stergiou et al. 2019):

(i) MAP: accuracy and precision greater than 5 mmHg
(ii) SAP: accuracy greater than 5 mmHg and precision greater than 8 mmHg

To assess the accuracy of the 3 technologies to measure changes in ABP, we measured the concordance rate which corresponded to the trend of changes before and after the introduction of vasopressor (Saugel et al. 2015).

Finally, the trend in each ABP component (SAP, MAP and SAP) during neuroradiological procedure will be analyzed using a mixed model analysis with patient-level random effect. For any comparison, statistical significance will be defined if *p*-value was above 0.05.

All statistical analysis was performed with R Statistical Software (version 3.6.1). Error grid analysis was performed using the open-access software designed by Saugel et al. (2018).

## Results

### Patient characteristics

From 18th March 2022 to 30th November 2022, 272 patients were eligible. Among them, 50 patients (15.4%) were included in the study. No statistical difference in baseline characteristics was found between included and excluded patients. Excluded patients were more frequently admitted in the operating room for thrombectomy (19.8% vs. 0%, *p* < 0.001) or diagnostic arteriography (58.6% vs. 6%, *p* < 0.001). Included patients were more frequently admitted for the treatment of SAH (12.2% vs. 38%, *p* < 0.001). An additional file illustrates the comparison between included and excluded patients [see Additional file 1]. Most patients were excluded due to the absence of invasive blood pressure monitoring during the procedure. Another subject was also excluded due to the impossibility of obtaining a valuable ABP curve on the Nexfin device. An additional file shows the flow diagram of the study [see Additional file 2]. Enrolled patients were mainly women (62%), with a mean age of 58 (± 12) years and were mostly ASA 2 (28%) or ASA 3 (60%). Half of the patients had hypertension or other cardiovascular comorbidities. Nearly all patients were admitted in the operating room for a cerebral embolization (94%) under general anaesthesia (96%). All embolization procedures were done for cerebral aneurysm securitization. Nineteen patients (38%) needed an emergency procedure for SAH. Baseline characteristics are summarized in Table 1.

**Table 1:** Baseline characteristics

	**Overall**
	***n* = 50**
Age (years)	58 (12)
Gender (men/women)	19/31
Weight (kg)	71.1 (16.5)
BMI (kg/m^2^)	25.2 (5.2)
Core temperature (°C)	36.5 (0.6)
SAPS II	23.8 (8.3)
Comorbidities	
Cardiovascular	25 (50)
Myocardial infarction	4 (8)
Arrhythmia	1 (2)
Hypertension	25 (50)
Arteritis	1 (2)
COPD	3 (6)
Diabetes	1 (2)
Chronic kidney disease	5 (10)
Medication	
Anti-hypertensive agents	20 (40)
Beta-blockers	2 (4)
ASA score	
1-2	31 (63.2)
3–5	19 (36.8)
Emergency procedure for SAH	19 (38)
Fisher score	
1-2	2 (10.6)
3	5 (26.3)
4	12 (63.2)
WFNS score	
1	14 (73.7)
2	1 (5.3)
4	2 (10.5)
5	2 (10.5)
Type of procedure	
Embolization	47 (94)
Other	3 (6)
General anaesthesia	48 (96)
Norepinephrine infusion	17 (34)

Data are given as *n* (%) or mean ± SD. *ASA* American Society of Anaesthesiologists, *BMI* body mass index, *COPD* chronic obstructive pulmonary disease, *SAH* sub-arachnoid haemorrhage, *SAPS* Simplified Acute Physiology Score, *SD* standard deviation, *WFNS* World Federation of Neurologic Surgeons

### Clinical concordance

**Fig. 1:**
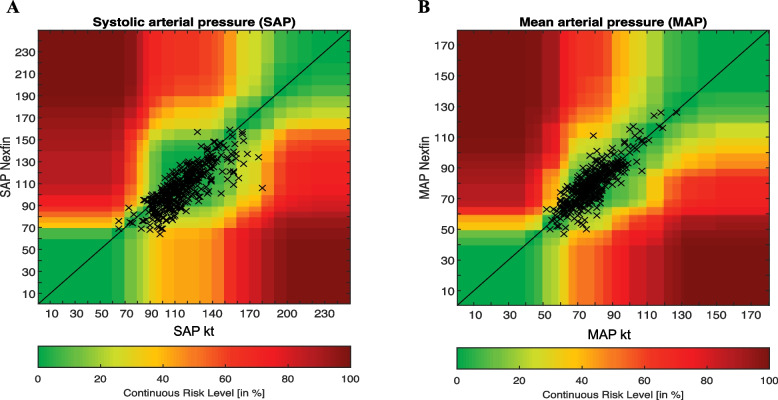
Error grid analysis heatmaps for SAP (**A**) and MAP (**B**) measurement with radial artery (Kt, gold standard) and non-invasive (Nexfin) device. MAP, mean arterial pressure; SAP, systolic arterial pressure

Compared to the arterial catheter, the error grid analysis revealed that 91.3% of SAP were in zone A, 8.1% were in zone B and 0.5% in zone C for the Nexfin (Fig. 1A). Considering the MAP, 86.1% of the measurement pairs were in zone A and 13.9% in zone B (Fig. 1B).

Overall, 38 invasive measures were characterized as hypertension events, and 46 were characterized as hypotension events (Mancia et al. 2023; Sessler et al. 2019). Among the 38 episodes of hypertension, 25 events (65.7%) were not detected by the Nexfin device. For these events, the mean SAP measured by Nexfin was about 126.2 mmHg (± 11). Among the 46 episodes of hypotensive events, 19 events (41%) were not detected by the Nexfin device. During these events, the mean MAP measured by Nexfin was about 70 mmHg (± 4.4).

### Accuracy of ABP measurements

**Fig. 2:**
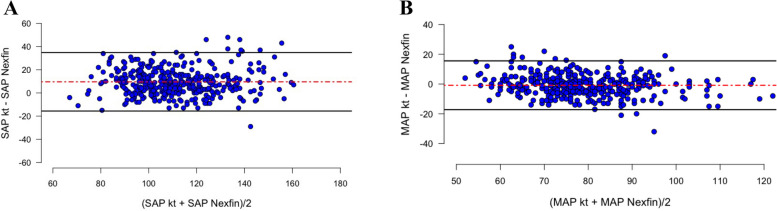
Bland-Altman analysis between ABP measurement (SAP and MAP) on invasive (radial artery, Kt) and non-invasive (Nexfin) devices. Each point corresponds to a pairs of measures. Red dashed lines represent mean bias. Black lines show the limit of agreement. **A** Accuracy and precision for SAP measures between Nexfin device and Kt. **B** Accuracy and precision for SAP measures between intermittent Arm Cuff and Kt. MAP, mean arterial pressure; SAP, systolic arterial pressure

In our dataset, we recorded 380 different paired ABP measurements with the Nexfin and the arterial catheter. Between the two devices (arterial catheter and the Nexfin), Bland-Altman analysis showed a mean bias of 9.6 mmHg (− 15.6 to 34.8 mmHg) for SAP and − 0.8 mmHg (− 17.2 to 15.6 mmHg) for MAP (Fig. 2A and B).

**Fig. 3:**
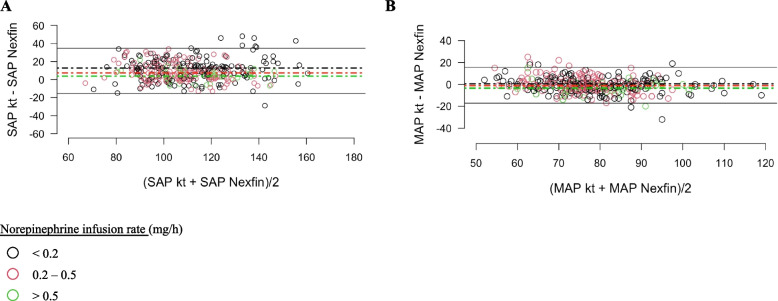
Bland-Altman analysis between ABP measurement (SAP and MAP) on invasive (radial artery, Kt) and non-invasive (Nexfin) devices divided into three sub-groups according to norepinephrine infusion rate: < 0.2 mg/h (black circle), from 0.2 to 0.5 mg/h (red circle) and > 0.5 mg/h (green circle). Black, red and green dashed lines representing mean bias respectively in each n sub-group: according to norepinephrine infusion rate: < 0.2 mg/h, from 0.2 to 0.5 mg/h and > 0.5 mg/h. **A** Accuracy and precision for SAP measures between Nexfin device and Kt in each sub-group. **B** Accuracy and precision for SAP measures between intermittent Arm Cuff and Kt in each sub-group. MAP, mean arterial pressure; SAP, systolic arterial pressure

For SAP, Bland–Altman analysis showed a mean bias of 12.8 mmHg (− 14.3 to 40 mmHg), 7.4 mmHg (− 13.1 to 28 mmHg) and 3.8 mmHg (− 10.3 to 18 mmHg) respectively for a norepinephrine infusion rate < 0.2 mg/h, from 0.2 to 0.5 mg/h and > 0.5 mg/h (Fig. 3A). For MAP, Bland-Altman analysis showed a mean bias of 0.4 mmHg (− 18.5 to 19.4 mmHg), − 1.3 mmHg (− 14.4 to 11.9 mmHg) and − 3.4 mmHg (− 17.4 to 10.5 mmHg) respectively for a norepinephrine infusion rate < 0.2 mg/h, from 0.2 to 0.5 mg/h and > 0.5 mg/h (Fig. 3B).

Compared to invasive measurements, the Nexfin device showed a good correlation for measures of SAP (*r*^2^ = 0.78, *p* < 0.001) and MAP (*r*^2^ = 0.82, *p* < 0.001) [see Additional file 3].

For DAP measurements, the two devices also showed a good correlation (*r*^2^ = 0.72, *p* < 0.001) [see Additional file 3]. Compared to arterial catheter, Bland-Altman analysis showed a mean bias of − 3.7 (− 19.9 to 12.5 mmHg) for the Nexfin. An additional file shows the Bland-Altman analysis of DAP obtained with invasive and non-invasive devices [see Additional file 4].

### Analysis of ABP changes during norepinephrine infusion

**Fig. 4:**
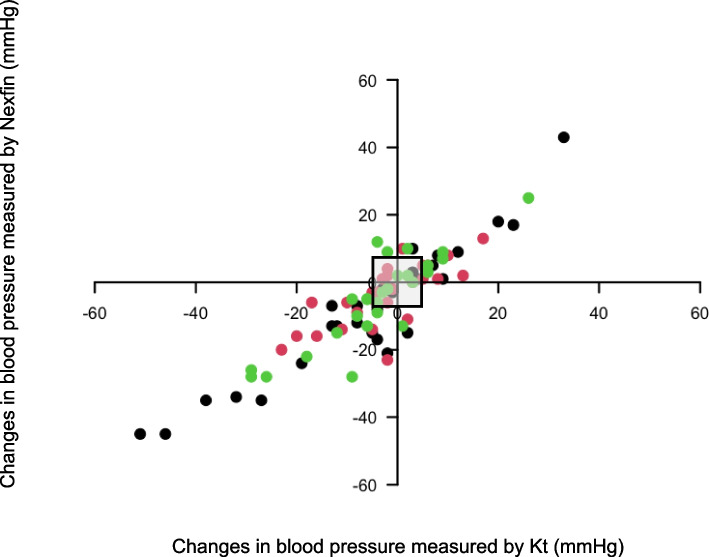
Four-quadrant plot with an exclusion zone of 5 mmHg (grey square) representing the trending in changes between invasive blood pressure and Nexfin blood pressure for SAP (black circle), DAP (red circle) and MAP (green circle). All these changes were recorded before and after the introduction of vasopressors during the procedure (30 pairs of measures were recorded for SAP, MAP and DAP). DAP, diastolic arterial pressure; MAP, mean arterial pressure; SAP, systolic arterial pressure

Compared to the ABP changes with the radial artery catheter (before and after the beginning of norepinephrine), the four-quadrant plot analysis showed a concordance rate of 92% for the ABP changes measured with the Nexfin (Fig. 4). This analysis comprised 84 pairs of measures.

### Evolution of ABP in each device

In comparison to radial artery catheter, SAP measures with Nexfin were significantly lower (*p* < 0.001). In comparison to radial artery catheter, MAP measures with Nexfin were not statistically different. In comparison to radial artery catheter, DAP measures with Nexfin were significantly higher (*p* < 0.001). An additional file illustrates the trend in each ABP parameter [see Additional file 5].

## Discussion

The main findings of the current observational study are as follows: (i) During an elective or emergent neuroradiological procedure, our error grid analysis showed that 99% of continuous non-invasive ABP measures obtained with Nexfin were located in risk zone A (‘no risk’) and risk zone B (‘low’ risk) regarding our error grid analysis (Saugel et al. 2018); (ii) With Nexfin, 41% of hypotensive and 65.7% of hypertensive events were not detected; (iii) Nexfin does not reach the AAMI criteria compare to invasive measures by artery catheter, considered the gold standard (Stergiou et al. 2019); (iv) With Nexfin, SAP tended to be overestimated using low dose of norepinephrine, while MAP tended to be lowered with the use of high dose of norepinephrine.

Previous studies have already investigated the accuracy of finger cuff technologies to measure ABP in the perioperative setting (Kim et al. 2014; Saugel et al. 2020). Only one study performed in the context of carotid endarterectomy demonstrated interchangeability with invasive measures (Heusdens et al. 2016). These findings and our results are in line with two meta-analyses (Kim et al. 2014; Saugel et al. 2020). In the most recent one, Saugel et al. (2020) found a pooled estimate for MAP bias of 4.19 with a limit of agreement from − 13.99 to 22.47 mmHg. In the same study, Saugel et al. (2020) also demonstrated that invasive and finger cuff technologies are not interchangeable for SAP and DAP measurements. Only 7% of the included studies found a complete accuracy between invasive and finger cuff technologies in regards to AAMI criteria (Saugel et al. 2020; Stergiou et al. 2019). In neurocritical care setting, one small observational study conducted in a stroke emergency department found a good correlation between non-invasive continuous blood pressure measured with the Nexfin device and standard measurement with arm cuff (Sen et al. 2014). During neuroradiological procedure, the studies are scarce (Bugarini et al. 2021). In this specific setting, only one smallest study was published in this setting concluding that Clearsight® is not interchangeable with invasive device in regards to AAMI criteria (Bugarini et al. 2021). Moreover, the number of exclusion criteria (such as advanced peripheral vascular disease, atrial fibrillation or peripheral oedema) limited the generalizability of their findings (Bugarini et al. 2021).

Although Bland-Altman statistic remains a key analysis to evaluate accuracy, it does not assess the clinical relevance of the maesurements compared to the gold standard (Saugel et al. 2018). The error grid analysis developed by Saugel et al. may help researchers to evaluate the clinical consequences in adopting an innovative device even if bias and limit of agreement are higher than recommended (Saugel et al. 2020). Only few studies have already evaluated the clinical consequences in adopting finger cuff technologies rather than invasive method to measure ABP in a perioperative setting (Bugarini et al. 2021; Schumann et al. 2021). During endarterectomy, ABP measurements with Nexfin instead of radial artery were not associated with an increase in proportion of patients outside a predefined ABP target before cross-clamping period (Heusdens et al. 2016). In obese patients, 77.1% and 89.5% of paired measures, respectively for MAP and SAP, were not associated with therapeutic consequences (Schumann et al. 2021). Moreover, they reported comparable results to our study as 99% of paired measures were located in the risk zone A (‘no’ risk) and risk zone B (‘low’ risk) in error grid analysis (Schumann et al. 2021). In the only published study in a neuroradiological setting, Bugarini et al. (2021) underlined that more than 85% of ABP paired measures were categorized in ‘no’ risk or ‘low’ risk zone, which is a percentage lower than our findings. The current study completes these findings by studying specifically hypotensive and hypertensive events. Our results tended to mitigate previous findings as a substantial amount of hypotensive and hypertensive events were not detected in our population (Bugarini et al. 2021; Schumann et al. 2021). Specific performances in extreme values were not reported in previous studies (Bugarini et al. 2021; Schumann et al. 2021). Moreover, we have also highlighted the impact of norepinephrine infusion rate that differentially affects SAP and MAP accuracy. In our population, DAP was the only measure that was poorly affected by norepinephrine infusion rate.

### Study implications

Our findings are in line with the results of the previous meta-analysis and found that the ABP measure with an invasive method is not strictly interchangeable with non-invasive devices (Kim et al. 2014; Saugel et al. 2020). Moreover, a substantial amount of hypotensive and hypertensive events were not detected by Nexfin in our population. However, according to our error grid analysis, we also found that the swap of invasive measurement to Nexfin might not be associated with any detrimental side effects or clinical drawback.

As arterial catheterization can be associated with potential harmful effects and may be a time-consuming procedure, the use of non-invasive device for ABP measurements might be a reliable alternative but needed further exploration to elucidate its use in daily practice. Moreover, Nexfin, like other finger cuff technologies, provides a continuous ABP measurement which needs further investigation and is probably relevant for high-risk neuroradiological procedure.

### Strength and limitations

The main strength of our study is the design as it is a prospective study. All consecutive patients were screened for eligibility, and screening information were described in the flowchart of the study. Moreover, we also included patients who needed emergency procedures which has not been done in other similar studies (Schumann et al. 2021). Data collections for ABP measures were standardized before the beginning of the study. This process was well-described in the study protocol and limits an evaluation bias. Lastly, we compared, in the same population, two different ways to measure ABP during a standardized interventional procedure.

Our study has also some limitations. It is a monocentric study. Furthermore, 43 eligible patients were not included in the final cohort, as research staff was unavailable on the day of the procedure. Thus, our study may suffer from selection bias, and our results might not be applicable to other settings. Second, the number of paired ABP measures is smaller compared to other studies (Bugarini et al. 2021). Our study is not a randomized controlled trial, and anaesthesiologists were not blinded to either devices. Furthermore, our study did not strictly report bias according to the AAMI standards. Indeed, the AAMI guidelines proposed to neglect any error in measurement when it falls within the ± 1 SD around the average value of the reference. Thus, these guidelines theoretically authorized a wider range of values than classically reported in method-comparison study with Bland-Altman analysis. However, this limitation was largely documented in previous studies, as shown in all studies included in the meta-analysis from Kim et al. (2014). Moreover, AAMI have edicted guidelines to evaluate new devices at rest and not in dynamic situation as encountered in the operating room. However, this methodology has been previously used to study dynamic changes (Kim et al. 2014). Finally, clinical concordance evaluated by error grid analysis, proposed by Saugel et al., was based on a consensus statement written by 25 experts. In this methodology, there are no strict thresholds which differentiate concordant versus nonconcordant ABP measures.

## Conclusion

During neuroradiological procedure, Nexfin is not strictly interchangeable with artery catheter regarding the AAMI guidance for ABP measuring. Nevertheless, the measurement of blood pressure with Nexfin seems to be a reliable alternative to invasive blood pressure monitoring during these procedures. Further studies are needed to delineate its clinical use in the operating room.

### Supplementary Information

Additional file 1. Comparison of patients’ characteristics between included and excluded patients.

Additional file 2. Flow diagram.

Additional file 3. Relationship between absolute values of ABP measurement (SAP, MAP and DAP) on invasive (radial artery, Kt) and non-invasive Nexfin. A: SAP measures with Nexfin and invasive method (380 pairs). B: MAP measures with Nexfin and invasive method (379 pairs). C: DAP measures with Nexfin and invasive method (381 pairs). ABP: Arterial Blood Pressure; MAP: Mean Arterial Pressure; SAP: Systolic Arterial Pressure.

Additional file 4. Bland-Altman analysis of diastolic arterial pressure (DAP) obtained with invasive (radial artery, kt) and Nexfin. A: Accuracy and precision for DAP measures between Kt and Nexfin. B: Accuracy and precision for DAP measures between Kt and Nexfin dividing in three sub-groups according to norepinephrine infusion rate: < 0.2 mg/h (black circle), from 0.2 to 0.5 mg/h (red circle) and > 0.5 mg/h (green circle). DAP: Diastolic Arterial Pressure.

Additional file 5. Evolution of SAP (panel A), MAP (panel B) and DAP (panel C) before the beginning and along the neuroradiological procedure obtained with the two devices.

## Data Availability

No datasets were generated or analysed during the current study.

## Abbreviations

AAMI: Association for the Advancement of Medical Instrumentation
ABP: Arterial blood pressure
DAP: Diastolic arterial pressure
LOA: Limits of agreement
MAP: Mean arterial pressure
SAH: Sub-arachnoid haemorrhage
SAP: Systolic arterial pressure
SD: Standard deviation
