# Supplementary material for: Continuous non-invasive vs. invasive arterial blood pressure monitoring during neuroradiological procedure: a comparative, prospective, monocentric, observational study
Source: Perioper Med (Lond). 2024 Jul 22;13:77. doi: 10.1186/s13741-024-00442-3 (PMC11265173; doi:10.1186/s13741-024-00442-3)
Supplement: Supplementary file 1 — Additional file 1. Comparison of patients’ characteristics between included and excluded patients. [file 13741_2024_442_MOESM1_ESM.docx]

**Additional file 1:** Comparison of patients’ characteristics between included and excluded patients

|  | **Inclusion** | |  |
| --- | --- | --- | --- |
|  | **No** | **Yes** | **p** |
|  | **n = 222** | **n = 50** |  |
| Age (years) | 61.3 (15.5) | 58.3 (12.3) | 0.2 |
| Gender (men) | 100 (45) | 19 (38) | 0.45 |
| Weight (kg) | 70.8 (15.4) | 71.1 (16.5) | 0.92 |
| BMI (kg/m^2^) | 25.3 (4.9) | 25.2 (5.2) | 0.97 |
| SAPS II | 28.6 (15.1) | 24.5 (7.8) | 0.4 |
| Comorbidities |  |  |  |
| Cardio-vascular | 102 (45.9) | 25 (50) | 0.72 |
| Myocardial infarction | 20 (9) | 4 (8) | 1 |
| Valvulopathy | 6 (2.7) | 0 (0) | 0.52 |
| Arrhythmia | 21 (9.5) | 1 (2) | 0.14 |
| Hypertension | 90 (40.5) | 25 (50) | 0.29 |
| Arteritis | 11 (5) | 1 (2) | 0.59 |
| COPD | 15 (6.8) | 3 (6) | 1 |
| Diabetes | 16 (7.2) | 1 (2) | 0.29 |
| Chronic kidney disease | 10 (4.5) | 5 (10) | 0.23 |
| Medication |  |  |  |
| Anti-hypertensive agents | 73 (32.9) | 20 (40) | 0.43 |
| Beta-blockers | 41 (18.5) | 2 (4) | 0.02 |
| ASA score |  |  | 0.67 |
| 1-2 | 132 (58.7) | 32 (63.3) |  |
| 3-5 | 90 (41.3) | 18 (36.7) |  |
| Reason for neuro radiological procedure |  |  |  |
| Emergency procedure for SAH | 27 (12.2) | 19 (38) | <0.001 |
| Fisher score |  |  | 0.42 |
| 1 | 3 (11.5) | 1 (5.3) |  |
| 2 | 4 (15.4) | 1 (5.3) |  |
| 3 | 3 (11.5) | 5 (26.3) |  |
| 4 | 16 (61.5) | 12 (63.2) |  |
| WFNS score |  |  | 0.27 |
| 1 | 13 (50) | 14 (73.7) |  |
| 2 | 7 (26.9) | 1 (5.3) |  |
| 4 | 3 (11.5) | 2 (10.5) |  |
| 5 | 3 (11.5) | 2 (10.5) |  |
| Stroke | 43 (19.4) | 0 (0) | 0.001 |
| Type of procedure |  |  | <0.001 |
| Arteriography | 130 (58.6) | 3 (6) |  |
| Embolization | 48 (21.6) | 47 (94) |  |
| Thrombectomy | 44 (19.8) | 0 (0) |  |
| General anaesthesia | 85 (38.3) | 48 (96) | <0.001 |

Data are given as n (%) or mean ± SD. ASA: American Society of Anaesthesiologists; BMI: Body Mass Index; COPD: Chronic Obstructive Pulmonary Disease; SAH: Sub-Arachnoid Haemorrhage; SAPS: Simplified Acute Physiology Score; SD: Standard Deviation; WFNS: World Federation of Neurologic Surgeons.
