# Supplementary material for: Continuous non-invasive vs. invasive arterial blood pressure monitoring during neuroradiological procedure: a comparative, prospective, monocentric, observational study
Source: Perioper Med (Lond). 2024 Jul 22;13:77. doi: 10.1186/s13741-024-00442-3 (PMC11265173; doi:10.1186/s13741-024-00442-3)
Supplement: Supplementary file 2 — Additional file 2. Flow diagram. [file 13741_2024_442_MOESM2_ESM.docx]

**Additional file 2:** Flow diagram

ABP: Arterial Blood Pressure; DAP: Diastolic Arterial Pressure; MAP: Mean Arterial Pressure; SAP: Systolic Arterial Pressure.
