# Supplementary material for: Continuous non-invasive vs. invasive arterial blood pressure monitoring during neuroradiological procedure: a comparative, prospective, monocentric, observational study
Source: Perioper Med (Lond). 2024 Jul 22;13:77. doi: 10.1186/s13741-024-00442-3 (PMC11265173; doi:10.1186/s13741-024-00442-3)
Supplement: Supplementary file 3 — Additional file 3. Relationship between absolute values of ABP measurement (SAP, MAP and DAP) on invasive (radial artery, Kt) and non-invasive Nexfin. A: SAP measures with Nexfin and invasive method (380 pairs). B: MAP measures with Nexfin and invasive method (379 pairs). C: DAP measures with Nexfin and invasive method (381 pairs). ABP: Arterial Blood Pressure; MAP: Mean Arterial Pressure; SAP: Systolic Arterial Pressure. [file 13741_2024_442_MOESM3_ESM.docx]

**Additional file 3:** Relationship between absolute values of ABP measurement (SAP, MAP and DAP) on invasive (radial artery, Kt) and non-invasive Nexfin. A: SAP measures with Nexfin and invasive method (380 pairs). B: MAP measures with Nexfin and invasive method (379 pairs). C: DAP measures with Nexfin and invasive method (381 pairs). ABP: Arterial Blood Pressure; MAP: Mean Arterial Pressure; SAP: Systolic Arterial Pressure.
