# Supplementary material for: Continuous non-invasive vs. invasive arterial blood pressure monitoring during neuroradiological procedure: a comparative, prospective, monocentric, observational study
Source: Perioper Med (Lond). 2024 Jul 22;13:77. doi: 10.1186/s13741-024-00442-3 (PMC11265173; doi:10.1186/s13741-024-00442-3)
Supplement: Supplementary file 4 — Additional file 4. Bland-Altman analysis of diastolic arterial pressure (DAP) obtained with invasive (radial artery, kt) and Nexfin. A: Accuracy and precision for DAP measures between Kt and Nexfin. B: Accuracy and precision for DAP measures between Kt and Nexfin dividing in three sub-groups according to norepinephrine infusion rate: < 0.2 mg/h (black circle), from 0.2 to 0.5 mg/h (red circle) and > 0.5 mg/h (green circle). DAP: Diastolic Arterial Pressure. [file 13741_2024_442_MOESM4_ESM.docx]

**Additional file 4:** Bland-Altman analysis of diastolic arterial pressure (DAP) obtained with invasive (radial artery, kt) and Nexfin. A: Accuracy and precision for DAP measures between Kt and Nexfin. B: Accuracy and precision for DAP measures between Kt and Nexfin dividing in three sub-groups according to norepinephrine infusion rate: < 0.2 mg/h (black circle), from 0.2 to 0.5 mg/h (red circle) and > 0.5 mg/h (green circle). DAP: Diastolic Arterial Pressure.
