# Supplementary material for: Continuous non-invasive vs. invasive arterial blood pressure monitoring during neuroradiological procedure: a comparative, prospective, monocentric, observational study
Source: Perioper Med (Lond). 2024 Jul 22;13:77. doi: 10.1186/s13741-024-00442-3 (PMC11265173; doi:10.1186/s13741-024-00442-3)
Supplement: Supplementary file 5 — Additional file 5. Evolution of SAP (panel A), MAP (panel B) and DAP (panel C) before the beginning and along the neuroradiological procedure obtained with the two devices. [file 13741_2024_442_MOESM5_ESM.docx]

**Additional file 5:** Evolution of SAP (panel A), MAP (panel B) and DAP (panel C) before the beginning and along the neuro radiological procedure obtained with the two devices
